# Current status and safety of laparoscopic surgery for patients with blunt abdominal trauma: A multicenter study using the Japan Trauma Data Bank

**DOI:** 10.1111/ases.13287

**Published:** 2024-02-07

**Authors:** Kenichiro Omoto, Chie Tanaka, Masamune Kuno, Shoji Yokobori

**Affiliations:** ^1^ Department of Emergency and Critical Care Medicine Nippon Medical School Tama Nagayama Hospital Tokyo Japan; ^2^ Department of Emergency and Critical Care Medicine Nippon Medical School Tokyo Japan

**Keywords:** blunt trauma, Japan Trauma Data Bank, laparoscopic surgery

## Abstract

**Introduction:**

This study was performed to examine current status and safety of laparoscopic surgery in adult patients with blunt trauma.

**Methods:**

Among 88 817 trauma patients registered in the Japan Trauma Data Bank from January 2019 to December 2022, we included blunt trauma patients who underwent laparoscopic surgery and/or laparotomy. We excluded patients with non‐blunt trauma, systolic blood pressure (sBP) of 0 mmHg on admission, age of <15 years, and Injury Severity Score (ISS) of 75. First, patients were divided into two groups: he laparoscopic surgery group (including patients transitioned from laparoscopic surgery) and the laparotomy‐only group. Second, missing values were estimated by multiple imputation. Finally, in‐hospital mortality was analyzed using propensity score matching to balance patient characteristics (age, sex, sBP, Glasgow coma scale (GCS) score, abdominal Abbreviated Injury Scale score, and ISS).

**Results:**

We analyzed 1301 patients [68 (5.2%) in the laparoscopic surgery group and 1233 (94.8%) in the laparotomy‐only group]. After propensity score matching, the in‐hospital mortality rate was 1.5% in the laparoscopy group and 10.0% in the laparotomy‐only group (*p* < .03). The odds ratio of in‐hospital mortality for the laparotomy‐only group after propensity score matching was 4.06 (95% confidence interval, 0.30–54.9; *p* = .29) compared with the laparoscopy group. After propensity score matching, there were no deaths occurred with patients of intra‐abdominal hemorrhage. In‐hospital mortality was not significantly different with peritonitis patients (*p* = .36).

**Conclusions:**

Laparoscopic surgical intervention was safely performed for selected adult patients with blunt abdominal trauma without an increase in mortality.

## 1 INTRODUCTION

The indications for surgical treatment of blunt abdominal trauma include peritonitis due to gastrointestinal perforation and intra‐abdominal hemorrhage, among others. Despite the advances in diagnostic modalities, accurate diagnosis of intraperitoneal injuries remains difficult,^1^ and the accuracy of diagnosis of gastrointestinal perforation with multidetector computed tomography is 82%–90%.^2^ Therefore, direct observation is necessary when the diagnosis is uncertain and a treatment plan cannot be determined. Exploratory laparotomy is commonly performed with a midline incision from the xiphoid process to the pubis.^3^ By contrast, laparoscopic surgery avoids the large wound that is necessary for laparotomy. Improvements in camera resolution, the development of various useful devices such as suturing instruments and hemostatic devices,^4^ and advances in surgeons' proficiency with the spread of laparoscopy have contributed to reliable confirmation of injuries and effective hemostasis and repair.

Therefore, laparoscopic surgery has enabled not only diagnosis but also repair. Emergency laparoscopic surgery for non‐traumatic gastrointestinal diseases has many advantages such as a smaller wound, shorter surgery time, reduced intraoperative bleeding, greater pain relief, and shorter duration of hospitalization.^5^ It has thus rapidly become a commonly used method. Nevertheless, the rate of laparoscopic surgery for abdominal trauma, including penetrating trauma, is about 5% in Japan,^6^ which is lower than the rate of 16% in the United Kingdom^7^ and 19% in the United States.^8^ Furthermore, only a few reports have described laparoscopic surgery for blunt trauma,^9^ and the level of evidence is low.^10^ Therefore, it is worthwhile to investigate the effectiveness of laparoscopic surgery. The probability of missing an injury to be repaired in laparoscopic surgery was previously reported to be unacceptably high,^11^ but it has recently been reported to be less than 1%, which is adequate for diagnostic capability.^12^ However, there are two prerequisites for safe laparoscopic surgery that differ from laparotomy. First, patients with head trauma must be carefully treated because the increase in intra‐abdominal pressure due to pneumoperitoneum may increase the intracranial pressure.^13^ Second, the patient must be hemodynamically stable.^12^ For these reasons, background factors should be adjusted when comparing laparoscopic surgery and laparotomy.

The aim of this study was to clarify the safety of laparoscopic surgical intervention in adults with blunt trauma based on in‐hospital mortality using the Japan Trauma Data Bank (JTDB).

## 2 METHODS

### 2.1 Study design and population

This study was approved by our hospital ethics committee (IRB No. 643). Of the 88 817 trauma patients enrolled in the JTDB from January 2019 to December 2022, 1301 patients who underwent abdominal surgery were retrospectively included in this study. We excluded patients with non‐blunt trauma, blood pressure of 0 mmHg at the time of admission, age of <15 years, and Injury Severity Score (ISS) of 75. To investigate the effect of laparoscopic surgery, we divided the patients into two groups: the laparoscopic group (including patients transitioned from laparoscopic surgery to laparotomy) and the laparotomy‐only group.

JTBD is a nationwide database in Japan that began registration in 2004, and 303 major hospitals are participating as of 2023. The following information is anonymized and accumulated: patient information (e.g., age, sex, time of injury, cause of injury, type of injury, injury mechanism), prehospital information (e.g., time, vital signs), information at emergency center (e.g., time, vital signs), imaging examinations (e.g., Focused Assessment with Sonography for Trauma (FAST), computed tomography), emergency procedures (e.g., endotracheal intubation, insertion of a chest tube, resuscitative endovascular balloon occlusion of the aorta (REVOA)), interventional radiology (IVR), transfusion, injury grade (Abbreviated Injury Scale (AIS) score by Site, ISS), diagnosis, surgery (time, indication, method), date of emergency department discharge, discharge date, and outcome.

### 2.2 Data collection

For selected patients, we collected data on age, sex, systolic blood pressure (sBP) and diastolic blood pressure on hospital arrival, respiratory rate on hospital arrival, body temperature on hospital arrival, Glasgow coma scale (GCS) score on hospital arrival, AIS score (head/neck, face, chest, abdomen, pelvis/extremities, and surface), ISS, FAST, blood transfusion within 24 h, IVR for abdominal region, abdominal surgical method, length of hospital stay, and in‐hospital mortality.

### 2.3 Statistical analysis

Continuous variables are presented as median (interquartile range), and nominal variables are presented as number and percentage. The *t*‐test was used to compare continuous variables, and the chi‐square test and Fisher's exact test were used to compare nominal variables.

We used six variables considered independent of mortality (age, sex, sBP, GCS score, abdominal AIS score, and ISS) to examine the association between laparoscopy and in‐hospital mortality in a multivariable analysis. For 4 missing variables (sBP, GCS score, abdominal AIS score, and ISS), we performed multiple imputation with 20 sets of data replaced by substitute values to reduce bias due to incomplete data, followed by propensity score matching to balance patient characteristics (age, sex, sBP, GCS score, abdominal AIS score, and ISS) between the two groups. Statistical significance was set at a *p*‐value of <.05. All statistical analyses were conducted using IBM SPSS version 23 (IBM Corp., Armonk, NY, USA).

## 3 RESULTS

The method of participant selection is shown in Figure 1. The 1301 patients were categorized into three groups: 56 (4.3%) who underwent laparoscopic surgery only, 12 (0.9%) who underwent laparoscopic surgery with conversion to laparotomy, and 1233 (94.8%) who underwent laparotomy only. To examine the impact of laparoscopic surgery, 68 (5.2%) patients who underwent laparoscopic surgery only and laparoscopic surgery with conversion to laparotomy were included in the laparoscopic surgery group. Analysis of the patients' background factors showed that the sBP on arrival and GCS score on arrival were significantly higher in the laparoscopic surgery group (*p* < .01 and *p* < .01, respectively) that and age, pulse rate on arrival, respiratory rate on arrival, and ISS were higher in the laparotomy‐only group (*p* < .01, *p* = .01, *p* = .03, and *p* < .01, respectively). After propensity score matching, sBP on arrival was significantly higher in the laparoscopic group (*p* = .02) and pulse rate on arrival was higher in the laparotomy‐only group (*p* = .02). The proportion of patients who received a blood transfusion within 24 h, who were FAST positive, who underwent IVR in the abdominal region, and the length of hospital stay were higher in the laparotomy‐only group (*p* < .01, *p* = .03, *p* < .01, *p* < .01 respectively).The mortality rate was 1.4% in the laparoscopy group and 18.9% in the laparotomy‐only group (*p* < .01). After propensity score matching, the proportion of patients who received a blood transfusion within 24 hours, who were FAST positive, who underwent IVR in the abdominal region, and the length of hospital stay were higher in the laparotomy‐only group (*p* < .01, *p* = .02, and *p* < .01, respectively). The in‐hospital mortality rate was 1.5% in the laparoscopy group and 10.0% in the laparotomy‐only group (*p* = .03) (Table 1). There was no statistically significant difference in the mechanism of injury between the two groups before and after propensity score matching was implemented (Table 2). Surgical indications were more in the laparotomy‐only group for both intra‐abdominal bleeding and peritonitis both before and after propensity score matching (*p* < .01 and *p* < .01, respectively) (Table 3).The odds ratio of in‐hospital mortality in the laparotomy‐only group compared with the laparoscopic surgery group was 4.06 (95% confidence interval, 0.30–54.9; *p* = .29) after propensity score matching (Table 4). Kaplan–Meier analysis showed that the laparoscopic surgery group had a significantly higher cumulative survival rate than the laparotomy‐only group (*p* = .02) (Figure 2). A subgroup analysis of 795 patients with intra‐abdominal bleeding and 250 patients with peritonitis showed that the in‐hospital mortality rate for intra‐abdominal bleeding was 0.0% in the laparoscopy group and 21.9% in the laparotomy‐only group (*p* = .02), with no deaths after propensity score matching (Table 5). The in‐hospital mortality rate in patients with peritonitis was 3.1% in the laparoscopy group and 12.5% in the laparotomy‐only group after propensity score matching, with no significant difference (*p* = .36) (Table 6).

**FIGURE 1:**
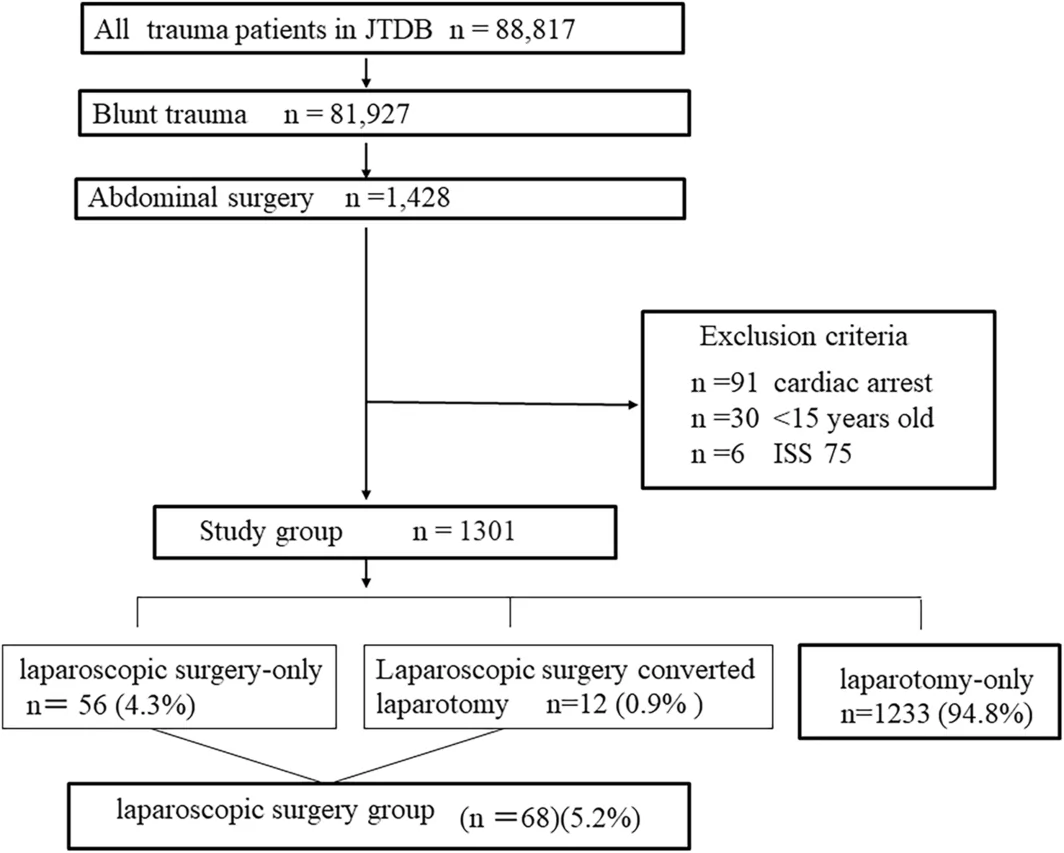
Flow diagram depicting study enrollment of patients with blunt trauma in the laparoscopic surgery group and laparotomy‐only group. JTDB, Japan Trauma Data Bank.

**TABLE 1:** Comparison of patients' demographic and prognosis between laparoscopic surgery group and laparotomy‐only group before and after propensity score‐matching analysis based on records from the Japan Trauma Data Bank.

	Before propensity score‐matching					After propensity score‐matching				
Variables	The laparoscopic surgery group (*n* = 68)		The laparotomy—only group (*n* = 1233)		*p*‐value	The laparoscopic surgery group (*n* = 67)		The laparotomy—only group (*n* = 67)		*p*‐value
Age	68/68	43 (26–69)	1233/1233	58 (37–73)	<.01	67/67	42 (20–64)	67/67	42 (20–64)	.94
Male	42/68	62	886/1233	72	.07	42/66	64	49/66	74	.20
sBP on arrival	67/68	124 (113–134)	1206/1233	111 (82–135)	<.01	67/67	124 (100–148)	67/67	110 (78–142)	.02
dBP on arrival	66/68	78 (68–89)	1124/1233	70 (55–86)	.07	66/67	78 (62–94)	64/66	70 (43–97)	.15
Pulse rate on arrival	67/68	83 (71–94)	1211/1233	94 (76–114)	.01	67/67	83 (62–104)	67/67	95 (69–121)	.02
Respiratory rate on arrival	66/68	20 (17–25)	1152/1233	24 (19–29)	.03	66/67	20 (13–27)	65/67	24 (16–32)	.19
Body temperature on arrival	65/68	36.8 (36.2–37.1)	1059/1233	36.3 (35.8–36.8)	<.01	64/67	36.8 (36.0–37.6)	57/67	36.4 (35.5–37.3)	.05
Glasgow coma scale on arrival	68/68	15 (14–15)	1190/1233	14 (12–15)	<.01	67/67	15 (13–17)	67/67	15 (12–18)	.07
AIS (head/neck)	11/68	0 (0–0)	308/1221	0 (0–1)	.02	67/67	0 (0–1)	67/67	0 (0–1)	.28
AIS (face)	4/68	0 (0–0)	112/1221	0 (0–0)	.70	67/67	0 (0–1)	67/67	0 (0–0)	.87
AIS (chest)	31/68	0 (0–3)	713/1221	2 (0–3)	.01	67/67	0 (0–2)	67/67	1 (0–3)	.58
AIS (abdomen)	63/68	3 (2–3)	1159/1221	3 (3–4)	<.01	67/67	3 (2–4)	67/67	3 (2–4)	.1
AIS (pelvis/extremities)	30/68	0 (0–2)	546/1221	0 (0–2)	.40	67/67	0 (0–1)	67/67	0 (0–2)	.55
AIS (surface)	22/68	0 (0–1)	325/1221	0 (0–1)	.65	67/67	0 (0–1)	67/67	0 (0–1)	.88
ISS	68/68	14 (9–20)	1213/1221	21 (13–29)	<.01	67/67	14 (5–23)	67/67	18 (7–29)	.72
BT within 24 h	21/67	31.3	866/1207	71.7	<.01	20/67	29.9	42/66	63.6	<.01
FAST positive	20/53	37.7	488/1050	46.5	.03	19/52	36.5	32/59	54.2	.06
IVR for abdominal region	4/67	6.0	250/1200	20.8	<.01	4/66	6.0	13/65	20.0	.02
Length of ICU stay, day	41/68	3 (1–5)	873/1233	56.4	.81	41/67	4 (0–8)	49/67	6 (3–15)	.09
Length of hospital stay, day	67/68	16 (9–22)	1161/1233	34.5	<.01	67/67	21 (4–38)	67/67	33 (5–60)	<.01
Mortality	1/68	1.4	233/1233	18.9	<.01	1/67	1.5	7/67	10.0	.03

*Note*: Data are provided as the total number and percentage of observations or as median (interquartile range).

Abbreviations: AIS, Abbreviated Injury Scale; BT, blood transfusion; dBP, diastolic blood pressure; FAST, focused assessment with sonography for trauma; ICU, intensive care unit; ISS, injury severity score; IVR, interventional radiology; sBP, systolic blood pressure.

**TABLE 2:** Comparison of frequency of mechanism of injury between laparoscopic surgery group and laparotomy‐only group.

	Before propensity score‐matching					After propensity score‐matching				
Variables	The laparoscopic surgery group (*n* = 68)		The laparotomy—only group (*n* = 1233)		*p*‐value	The laparoscopic surgery group (*n* = 67)		The laparotomy—only group (*n* = 67)		*p*‐value
Motor vehicle crash	54/68	79.4	898/1231	72.8	.23	53/67	79.1	52/67	77.6	.83
Fall	10/68	14.7	219/1231	17.8	.52	10/67	14.9	11/67	16.4	.81
Impact from objects or people	2/68	2.9	66/1231	5.4	.58	2/67	3.0	3/67	4.5	1.00
Crushing	1/68	1.5	34/1231	2.8	1.00	1/67	1.5	0/67	0.0	1.00
Train	1/68	1.5	4/1231	0.3	.24	1/67	1.5	0/67	0.0	1.00
Machinery	0/68	0.0	9/1231	0.7	1.00	0/67	0.0	1/67	1.5	1.00
Natural disaster	0/68	0.0	1/1231	0.0	1.00	0/67	0.0	0/67	0.0	NA

*Note*: Analysis based on records from the Japan Trauma Data Bank. Data are provided as the total number and percentage of observations.

Abbreviation: NA, not applicable.

**TABLE 3:** Comparison of frequency of surgical indications between laparoscopic surgery group and laparotomy‐only group before and after propensity score‐matching analysis based on records from the Japan Trauma Data Bank.

	Before propensity score‐matching					after propensity score‐matching				
Variables	The laparoscopic surgery group (*n* = 68)		The laparotomy—only group (*n* = 1233)		*p*‐value	The laparoscopic surgery group (*n* = 67)		The laparotomy—only group (*n* = 67)		*p*‐value
Intra‐abdominal hemorrhage	18/68	26.5	777/1233	63.0	<.01	17/63	27.0	38/65	58.5	<.01
Peritonitis	32/68	47.1	218/1233	17.7	<.01	32/63	50.8	18/65	27.7	<.01
Retroperitoneal hemorrhage	2/68	2.9	68/1233	5.5	.58	2/63	3.2	3/65	4.6	.67
Bladder rupture	2/68	2.9	6/1233	0.0	.06	2/63	3.2	1/65	1.5	.54
Open abdominal wall wound	0/68	0.0	27/1233	0.0	.40	0/63	0.0	2/65	3.1	.16
ACS	0/68	0.0	18/1233	0.0	.62	0/63	0.0	0/65	0.0	NA
Others	21/64	32.8	264/1233	22.4	.07	21/63	33.3	13/65	20.0	.09

*Note*: Analysis based on records from the Japan Trauma Data Bank. Data are provided as the total number and percentage of observations.

Abbreviations: ACS, abdominal compartment syndrome; NA, not applicable.

**TABLE 4:** Regression analysis for risk of mortality, adjusted for laparoscopic surgery and other confounding factors, in patients with blunt trauma.

	Before propensity score‐matching			After propensity score‐matching		
	Odds ratio	95%CI	*p*‐value	Odds ratio	95%CI	*p*‐value
Laparotomy	5.64	0.74–43.0	.10	4.06	0.30–54.9	.29
Laparoscopic surgery (reference)	1			1		
Age	1.03	1.02–1.04	<.01	1.04	0.99–1.10	.13
Male	1.30	0.87–1.96	.19	0.70	0.09–5.62	.74
Female (reference)	1			1		
sBP on hospital arrival	0.99	0.99–1.00	<.01	0.97	0.94–1.00	.32
GCS on hospital arrival	0.82	0.79–0.86	<.01	0.84	0.62–1.12	.23
AIS (abdomen)	0.87		.11	0.55	0.23–1.35	.19
ISS	1.03	1.01–1.04	<.01	1.11	1.00–1.24	.05

Abbreviations: CI, confidence interval; FAST, focused assessment with sonography for trauma; GCS, Glasgow Coma Scale; ISS, injury severity score; sBP, systolic blood pressure.

**FIGURE 2:**
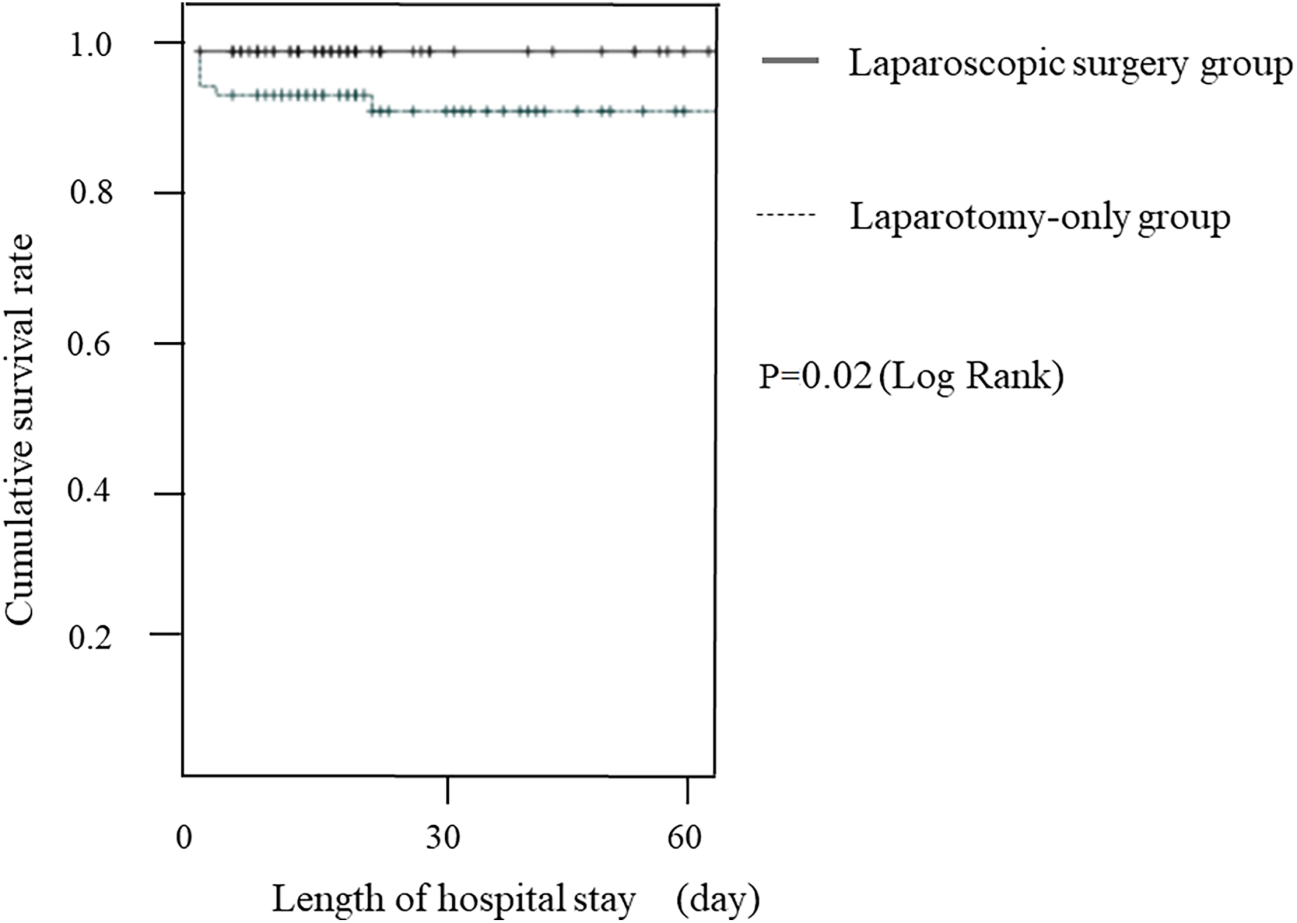
Kaplan–Meier analysis between the laparoscopic surgery group and laparotomy‐only group.

**TABLE 5:** Comparison of patients' demographic and prognosis which the indication for surgery is intra‐abdominal bleeding between laparoscopic surgery group and laparotomy‐only group before and after propensity score‐matching analysis based on records from the Japan Trauma Data Bank.

	Before propensity score‐matching					After propensity score‐matching				
Variables	The laparoscopic surgery group (*n* = 18)		The laparotomy—only group (*n* = 777)		*p*‐value	The laparoscopic surgery group (*n* = 17)		The laparotomy—only group (*n* = 17)		*p*‐value
Age	18/18	46 (28–63)	777/777	57 (36–78)	.01	17/17	43 (25–61)	17/17	36 (22–50)	.63
Male	11/18	61	566/776	72.9	.29	11/17	64.7	12/17	70.6	1.00
sBP on arrival	17/18	119 (87–151)	756/777	105 (68–142)	.10	17/17	119 (87–151)	17/17	111 (72–150)	.55
dBP on arrival	17/18	78 (62–94)	694/777	67 (41–94)	.18	17/17	78 (62–94)	16/17	76 (51–101)	.54
Pulse rate on arrival	17/18	83 (64–102)	763/777	96 (69–123)	.13	17/17	83 (64–102)	17/17	86 (56–117)	.35
Respiratory rate on arrival	17/18	20 (14–26)	724/777	24 (15–33)	.06	17/17	20 (14–26)	15/17	24 (13–35)	.07
Body temperature on arrival	17/18	36.8 (36.1–37.5)	651/777	36.2 (35.2–37.2)	.02	16/17	36.8 (36.1–37.5)	16/17	36.5 (35.8–37.2)	.02
Glasgow coma scale on arrival	18/18	14 (13–15)	750/777	14 (10–18)	.02	17/17	14 (13–15)	17/17	15 (13–17)	.31
AIS (head/neck)	18/18	0 (0–1)	769/777	0 (0–1)	.13	17/17	0 (0–1)	17/17	0 (0–1)	.80
AIS (face)	18/18	0 (0–0)	769/777	0 (0–1)	.59	17/17	0 (0–0)	17/17	0 (0–1)	.52
AIS (chest)	18/18	1 (0–2)	769/777	2 (0–4)	.16	17/17	0 (0–1)	17/17	0 (0–2)	1.00
AIS (abdomen)	18/18	3 (2–4)	769/777	3 (3–4)	<.01	17/17	3 (2–4)	17/17	3 (2–4)	.74
AIS (pelvis/extremities)	18/18	1 (0–2)	769/777	0 (0–2)	.70	17/17	0 (0–1)	17/17	0 (0–1)	.39
AIS (surface)	18/18	0 (0–1)	769/777	0 (0–1)	.25	17/17	0 (0–1)	17/17	0 (0–0)	.55
ISS	18/18	15 (7–22)	764/777	24 (11–37)	<.01	17/17	16 (8–24)	17/17	16 (6–26)	.70
BT within 24 h	10/17	58.8	650/763	85.2	.02	9/16	56.3	13/16	81.3	.28
FAST positive	8/13	61.5	443/679	65.2	.78	7/12	58.3	13/16	92.9	.23
IVR for abdominal region	3/18	16.7	183/760	24.1	.59	1/17	5.9	3/17	29.4	.60
Length of ICU stay, day	8/18	3 (0–5)	560/777	5 (0–1700)	.90	8/17	3 (0–5)	12/17	2 (0–4)	.25
Length of hospital stay, day	18/18	14 (0–29)	729/777	23 (0–61)	.06	17/17	16 (1–31)	17/17	11 (0–42)	.11
Mortality	0/18	0.0	170/777	21.9	.02	0/17	0.0	0/17	0.0	NA

*Note*: Data are provided as the total number and percentage of observations or as median (interquartile range).

Abbreviations: AIS, Abbreviated Injury Scale; BT, blood transfusion; dBP, diastolic blood pressure; FAST, focused assessment with sonography for trauma; ICU, intensive care unit; ISS, injury severity score; IVR, interventional radiology; sBP, systolic blood pressure.

**TABLE 6:** Comparison of patients' demographic and prognosis which the indication for surgery is peritonitis between laparoscopic surgery group and laparotomy‐only group before and after propensity score‐matching analysis based on records from the Japan Trauma Data Bank.

	Before propensity score‐matching					After propensity score‐matching				
Variables	The laparoscopic surgery group (*n* = 32)		The laparotomy—only group (*n* = 218)		*p*‐value	The laparoscopic surgery group (*n* = 32)		The laparotomy—only group (*n* = 32)		*p*‐value
Age	32/32	57 (37–76)	218/218	61 (40–82)	.40	32/32	57 (37–76)	32/32	49 (27–71)	.72
Male	22/32	68.8	155/217	71.4	.76	10/32	31.3	10/32	31.3	1.00
sBP on arrival	32/32	125 (106–144)	217/218	122 (88–156)	.48	32/32	125 (106–144)	32/32	142 (104–179)	.30
dBP on arrival	31/32	79 (62–96)	210/218	76 (54–98)	.74	31/32	79 (62–96)	31/32	82 (58–106)	.18
Pulse rate on arrival	32/32	82 (63–100)	217/218	88 (64–112)	.08	32/32	82 (63–100)	32/32	90 (69–111)	.04
Respiratory rate on arrival	32/32	21 (14–27)	208/218	23 (16–30)	.44	32/32	21 (14–27)	30/32	21 (12–29)	.60
Body temperature on arrival	31/32	36.8 (35.9–37.7)	205/218	36.5 (35.4–37.6)	.31	31/32	36.8 (35.9–37.7)	31/32	36.4 (35.3–37.5)	.23
Glasgow coma scale on arrival	32/32	15 (13–15)	210/218	15 (13–17)	.70	32/32	15 (13–15)	32/32	15 (13–15)	.75
AIS (head/neck)	32/32	0 (0–1)	217/218	0 (0–1)	.13	32/32	0 (0–1)	32/32	0 (0–1)	.63
AIS (face)	32/32	0 (0–0)	217/218	0 (0–0)	.17	32/32	0 (0–0)	32/32	0 (0–1)	.09
AIS (chest)	32/32	0 (0–2)	217/218	0 (0–2)	.56	32/32	0 (0–2)	32/32	0 (0–1)	.86
AIS (abdomen)	32/32	3 (3–3)	217/218	3 (2–4)	.84	32/32	3 (3–3)	32/32	3 (3–3)	.57
AIS (pelvis/extremities)	32/32	0 (0–1)	217/218	0 (0–1)	.88	32/32	0 (0–1)	32/32	0 (0–1)	.34
AIS (surface)	32/32	0 (0–0)	217/218	0 (0–0)	.25	32/32	0 (0–0)	32/32	0 (0–1)	1.00
ISS	32/32	13 (6–20)	217/218	17 (7–27)	.18	32/32	13 (6–20)	32/32	14 (5–22)	.90
BT within 24 h	5/31	15.6	88/216	47.7	<.01	5/32	15.6	10/32	31.3	.24
FAST positive	8/32	25.0	72/179	40.2	.36	8/26	30.8	11/26	42.3	.57
IVR for abdominal region	1/32	3.1	26/214	12.1	.22	1/32	3.1	6/31	19.4	.05
Length of ICU stay, day	19/32	2 (0–4)	144/218	3 (0–18)	.17	19/32	2 (0–4)	23/32	3 (0–11)	.20
Length of hospital stay, day	31/32	17 (0–34)	210/218	23 (0–54)	.02	31/32	17 (0–37)	29/32	25 (0–63)	.04
Mortality	1/32	3.1	15/218	6.9	.70	1/32	3.1	4/32	12.5	.36

*Note*: Data are provided as the total number and percentage of observations or as median (interquartile range).

Abbreviations: AIS, Abbreviated Injury Scale; BT, blood transfusion; dBP, diastolic blood pressure; FAST, focused assessment with sonography for trauma; ICU, intensive care unit; ISS, injury severity score; IVR, interventional radiology; sBP, systolic blood pressure.

## 4 DISCUSSION

Lower short‐term postoperative mortality rates have been reported in the laparoscopic surgery group for distal gastric cancer,^14^ colorectal liver metastases,^15^ major liver resection,^16^ transverse colon cancer,^17^ colon cancer,^18^ and perforated peptic ulcer.^19^ Unlike these conditions, postoperative short‐term mortality for trauma cannot be simply compared. This cohort study was performed to determine the safety of laparoscopic surgery versus laparotomy alone in adult patients with blunt abdominal trauma as documented by the Japanese National Trauma Registry. Our data showed that in‐hospital mortality complemented the missing values and did not increase after adjustment for confounders by propensity score matching, indicating that laparoscopic surgery was safely performed even in blunt abdominal trauma. Additionally, subgroup analysis was performed because hemorrhage and peritonitis may require different treatment strategies depending on the indication for surgery. There were no deaths associated with laparoscopic surgery, that laparoscopic surgery may have been performed in only in patients at low risk of death. In peritonitis, there was no difference in mortality between the laparoscopic surgery group and the laparotomy‐only group, indicating that laparoscopic surgery was performed safely.

The strength of this study is that it is based on records from a nationwide multicenter survey with further adjustment for confounding factors. Although previous studies comparing laparoscopic surgery and laparotomy alone in patients with blunt trauma have shown no significant difference in in‐hospital mortality,^20^, ^21^ evidence is inadequate, with possible bias due to small amounts of data and single‐center analyses. Studies using national databases in Japan^6^ and the United States^8^ showed no significant difference in mortality between laparoscopic surgery and laparotomy alone for abdominal trauma as a whole, but these studies included cases other than blunt trauma and did not adjust for confounding factors when comparing the two treatment methods. Therefore, this is the first report to demonstrate the effectiveness of laparoscopic surgery for blunt trauma in adult patients using a nationwide database.

This study also has several limitations. First, it is a retrospective, database‐based study. Second, the limited number of centers available for laparoscopic emergency surgery may have introduced bias. Third, the skill level of surgeons and facilities performing laparoscopic procedures is unknown. Fourth, the small size of the laparoscopic surgery group limits the number of coordination factors. Finally, unmeasured confounders may have influenced the results.

## 5 CONCLUSION

Laparoscopic surgical intervention was safely performed for selected adult patients with blunt abdominal trauma without an increase in mortality.

## CONFLICT OF INTEREST STATEMENT

None declared.

## ETHICS STATEMENT

The study protocol was approved by the Ethics Committee of Nippon Medical School Tama Nagayama Hospital (IRB No. 643). The requirement for informed consent from patients was waived.

## Data Availability

The data that support the findings of this study are available from Japan Trauma Data Bank. Restrictions apply to the availability of these data, which were used under license for this study. Data are available from the author(s) with the permission of Japan Trauma Data Bank.
